# Differences in Stereoscopic Luster Evoked by Static and Dynamic Stimuli

**DOI:** 10.1177/2041669519846133

**Published:** 2019-05-17

**Authors:** Gunnar Wendt, Franz Faul

**Affiliations:** Institut für Psychologie, Christian-Albrechts-Universität zu Kiel, Germany

**Keywords:** color, binocular vision, lightness or brightness, surfaces or materials

## Abstract

We compared the classic static stereoscopic luster phenomenon with a recently described dynamic variant (“counter modulation”) to investigate whether they are related to the same or different processes. In the experiments, we presented pairs of center-surround stimuli haploscopically and measured the effect of the contrast between center colors on perceived luster. The center colors were either static or temporally modulated. In addition, we examined five color conditions (one achromatic, two equiluminant, and two mixed conditions) and three background conditions that influence the channel-wise polarities of the contrast of the two centers to the common surround. The results for static and dynamic stimuli differed in several ways, suggesting that they depend on different mechanisms: Compared with the static version, in dynamic stimuli, luster was perceived at markedly lower contrasts, did not depend on the sign of the contrast polarities, and appeared more steady. However, both phenomena seem also similar in some respects: In both cases, equiluminant stimuli led to lustrous impressions that were considerably less strong than those evoked by stimuli containing luminance variation, and the strength of the perceived luster was generally boosted with reversed contrast polarities.

## Introduction

**Figure 1.**
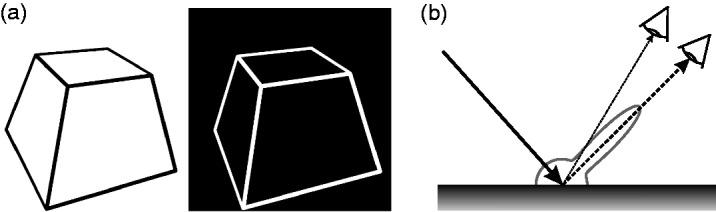
(a) Two perspective line drawings of a truncated pyramid with inverted intensities similar to the stimuli used by Dove (1851). The stereoscopic fusion of these two half-images makes the stimulus appear lustrous (stimulus is arranged for uncrossed viewing). (b) The reflective behavior of a glossy surface can be described by the BRDF—the bidirectional reflectance distribution function (Nicodemus, Richmond, Hsia, Ginsberg, & Limperis, 1977). Since the specular component (represented by the specular lobe) is directionally selective, the two eyes of an observer generally receive different amounts of light from the same surface point (dashed arrows).

When in the middle of the 19th century Heinrich Dove (1851) discovered the phenomenon of stereoscopic luster, this established the research field of material perception (Adelson, 2001; Fleming, 2014). Dove used two perspective line drawings of a truncated pyramid with inverted intensities and found that the faces of this geometric body yielded a lustrous appearance when the two images were haploscopically fused by means of a stereoscope (Figure 1(a)). This discovery triggered the interest of many other researchers who offered different explanations for this phenomenon (for a more detailed overview, see Mausfeld, Wendt, & Golz, 2014).

One approach assumed that the lustrous appearance was the result of some sort of conflict at a physiological level—a response of the visual system indicating its inability to combine these two discrepant monocular intensity signals (Brewster, 1861; Dove, 1851, 1859; Rood, 1861). In contrast, Oppel (1854) and von Helmholtz (1867) attributed a functional meaning to this phenomenon. They assumed that it results from a mechanism of the visual system that exploits a physical regularity of light reflection: As a first approximation, the reflective behavior of a glossy surface can be described as a combination of an ideal diffuse (Lambertian) and a specular reflection. The magnitude of the diffuse component depends only on the angle between the surface normal and the direction to the light source, whereas the specular component also depends on the viewing direction. As a consequence of the latter property, left and right eye in general receive different amounts of reflected light from the same point of a glossy surface, because the viewing directions of the two eyes differ slightly (Figure 1(b)). Thus, whenever different luminances occur at corresponding retinal areas, the visual system may infer that this is caused by light reflected from a glossy surface (Jung, Moon, Park, & Song, 2013). Note that in the following, we will use the terms luster and gloss interchangeably since it is at present not clear how these two subjectively similar phenomena relate to each other—it is the main aim of this study to examine this relationship more closely. From this view, the phenomenon of stereoscopic luster demonstrates the role of binocular cues for the perception of glossiness (Blake & Bülthoff, 1990; Mausfeld et al., 2014; Sakano & Ando, 2010; Wendt, Faul, Ekroll, & Mausfeld, 2010; Wendt, Faul, & Mausfeld, 2008). The Oppel–Helmholtz interpretation has been widely accepted by subsequent researchers in this field (e.g., Brücke, 1861; Bühler, 1922).

However, the functionalistic interpretation of Oppel and Helmholtz was challenged by findings from Anstis (2000). A first critical observation was that reversed contrast polarities in the two monocular stimuli were crucial for a lustrous appearance: In his flat center-surround stimuli, strong lustrous impressions occurred only when a spatial decrement in one eye was paired with a spatial increment in the other (“inc-dec pairing,” i.e., when one center patch had a lower luminance than its surround, the other a higher luminance). It is unclear how this condition can be related to an ecological gloss situation: If the surround is interpreted as the diffuse component of a glossy surface and the central patches as locations from which light is reflected specularly to the observer, then the central patches should never be decremental, because the specular component always adds light. A second critical observation was that a very similar effect could also be obtained under monocular viewing conditions, where the two single stimuli were presented alternately at a flicker rate of 16 Hz (for another interesting method to produce a lustrous effect with a static monocular stimulus, see Pinna, Spillmann, & Ehrenstein, 2002). These results obviously challenge an interpretation in terms of a binocular cue that relates to a physical regularity of surface reflectance and instead support the alternative interpretation that ascribes stereoscopic luster (as well as monocular luster from flicker) to a neuronal conflict. More specifically, Anstis (2000) interprets this conflict as a competition between ON and OFF visual pathways (Schiller, 1992), activated by spatially incremental or decremental light patterns, respectively (see also Burr, Ross, & Morrone, 1986; Georgeson, Wallis, Meese, & Baker, 2016; Pinna et al., 2002).

**Figure 2.**
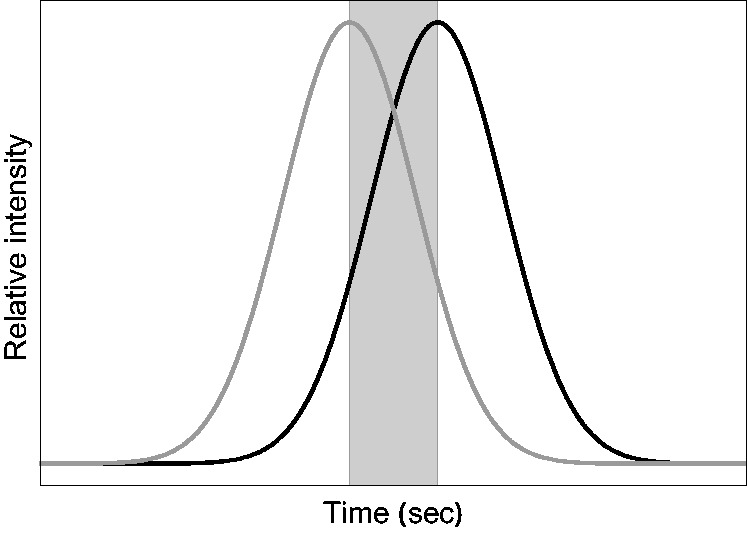
In a former study (Mausfeld et al., 2014), two haploscopically presented center-surround stimuli were used where the luminances of the center patches changed according to a Gaussian function. The two intensity functions were temporally shifted between the left eye (gray curve) and the right eye (black curve) by 150 ms. The shaded area shows the time interval during which a temporal decrement in the left eye was combined with a temporal increment in the right eye.

**Figure 3.**
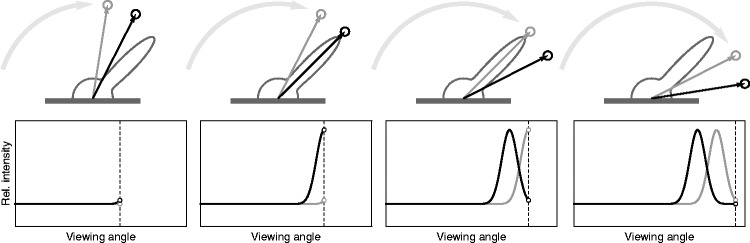
When an observer moves around a glossy object while fixating a certain point on the surface, the reflected light will produce overlapping intensity functions in the two eyes.

In a recent article, a new variant of stereoscopic luster was described that is evoked by center-surround stimuli in which the luminances of the center patches are temporally modulated according to a Gaussian function (Mausfeld et al., 2014). The Gaussian intensity functions in the two monocular half-images were shifted in time so that the right eye received the intensity peak 150 ms later than the left eye (Figure 2). This led to strong impressions of luster if the width of the Gaussians was chosen properly. Contrary to what Anstis (2000) found with his stimuli, the impression of luster elicited by such dynamic stimuli was unaffected by changes in the surround luminance. In particular, a spatial inc-dec pairing was not necessary for the effect. It was found that the lustrous impression occurred within a time interval during the stimulus presentation that was located between the two peaks of the Gaussians, that is, where a *temporal* decrement (i.e., a decreasing intensity curve) in one center was accompanied by a *temporal* increment in the other center (i.e., an increasing curve; see the shaded area in Figure 2). This counter modulation may actually serve as a dynamic binocular cue for glossiness. As Figure 3 illustrates, similar temporal intensity functions are produced by real gloss situations, for instance, when an observer moves around a glossy object while fixating a certain point on its surface.

Further investigations with this type of stimuli showed that the presence of counter modulation in itself is not sufficient to generate luster: If the intensity baseline of the temporal intensity functions for both eyes was increased relative to the modulation phase, so that the baseline did no longer constitute the lowest luminance, then the impression of luster gradually diminished with increasing baseline intensity. This observation also seems to be in agreement with physical regularities: The bottom graphs in Figure 3 show that the level of the constant intensity baseline is determined by the diffuse component of the surface. As mentioned earlier, the amount of light that is produced by the specular component will always add to this base level. Therefore, intensity modulations below this baseline would be physically implausible—which could explain why the lustrous sensations rapidly diminished when the baseline was increased.

In this brief summary of previous findings, we contrasted three different methods to elicit phenomenal luster, namely, static binocular stimuli, alternating monocular presentation, and counter modulation in dynamic binocular stimuli, and two different interpretations of the resulting phenomena, namely, a relatively low-level conflict hypothesis and a functionalistic hypothesis that relates binocular luster to normal gloss perception. Neither of the two hypotheses can fully explain the observations made so far: The conflict hypothesis can account for the observations made with static binocular and monocular stimuli, but fails to explain the occurrence of luster in dynamic binocular stimuli, because in this case, incompatible spatial contrast polarities, on which this explanation rests, are not necessary for the effect. The functional explanation, on the other hand, seems in line with the findings made with the dynamic binocular stimuli. However, the role of the spatial contrast polarity that influences luster in static binocular stimuli remains unclear in this approach, and for obvious reasons, it can also not explain the occurrence of monocular luster. A possible explanation of this state of affairs could be that both hypotheses are wrong or incomplete. Alternatively, one may assume that the similarity of the phenomenal impressions elicited in the three experimental situations is deceptive and conceals the fact that they actually have different causes. This does not exclude the possibility that several factors contribute in a given situation.

### The General Approach Used in the Current Experiments

In the present article, we report the results of a number of experiments with which we investigated the plausibility of the assumption that static and dynamic stimuli trigger different mechanisms. Our main strategy was to compare the strength of lustrous effects produced by the two binocular stimuli (i.e., classical static binocular stimuli vs. counter-modulation stimuli) under several color conditions that differ with respect to their physical plausibility (considered relevant by the functionalistic hypothesis) and at the same time systematically influence the spatial contrast polarities (considered relevant in the conflict hypothesis).

The control of the polarities of the spatial contrast between center and surround in the experiments is essential, because they play a central role in the conflict hypothesis. If the phenomenon of stereoscopic luster is actually due to a neuronal conflict between discrepant signals from corresponding visual pathways, luster should only be observed when the contrast polarities in the two monocular half-images are reversed. We used stimuli in which the contrast polarity in each of the three color channels was either identical or reversed by choosing appropriate colors for center and surround. In this way, we were able to compare the strength of perceived luster with and without conflict.

At the same time, we investigated five different color conditions that determined the binocular color difference between the center patches of the two half-images. Each color condition was related to an axis in color space. Besides isolated variations in luminance along a purely achromatic axis, we also examined two equiluminant chromatic axes and two mixed axes with simultaneous variations in chromaticity and luminance. From the physical perspective underlying the hypothesis of Oppel and Helmholtz, the five color conditions are not all equally plausible in realistic gloss situations. Although some glossy materials exist, whose chromatic properties vary to some extent with changing angles between surface normal, light source direction, and viewing direction (e.g., certain kinds of fabric, such as shot silk or changeable taffeta, see Lu, Koenderink, & Kappers, 2000), it is obviously not this chromatic variability that is responsible for the perceived glossiness. Thus, from this perspective, the two equiluminant color conditions are presumably the most unrealistic ones in our study and should lead to comparatively weaker lustrous sensations. Perceived glossiness is usually associated with differences in luminance, since, as mentioned earlier, the light that is reflected in a specular manner from a surface always adds to the diffusely reflected light. The three remaining color conditions include luminance variations and should therefore be more likely to evoke lustrous impressions. The two mixed color conditions comprise also variations in chromaticity. If the underlying mechanism of the visual system is exclusively sensitive to luminance information and ignores chromatic information, this should make no difference.

## Methods

### General Construction of the Stimuli

Pairs of center-surround stimuli were haploscopically fused by means of a mirror stereoscope (ScreenScope, Monitor Version). The stimuli were presented on a TFT monitor (EIZO CG243W) with a screen width of 52 cm and a screen height of 32.5 cm (image resolution: 1920 by 1200 pixels). The monitor was calibrated according to a standard procedure as described in Brainard (1989) using a JETI specbos 1211 spectroradiometer. The center patch of each monocular half-image was a square with a side length of 3.67° of visual angle, and it was embedded in a common background that filled the entire screen. To facilitate the fusion of the stimuli, the center regions in both monocular half-images were flanked by one-pixel thick right angles near each corner with a side length of 1.95° of visual angle in all experiments. Depending on the luminance of the background, these fusion locks either appeared in a white or black color.

**Figure 4.**
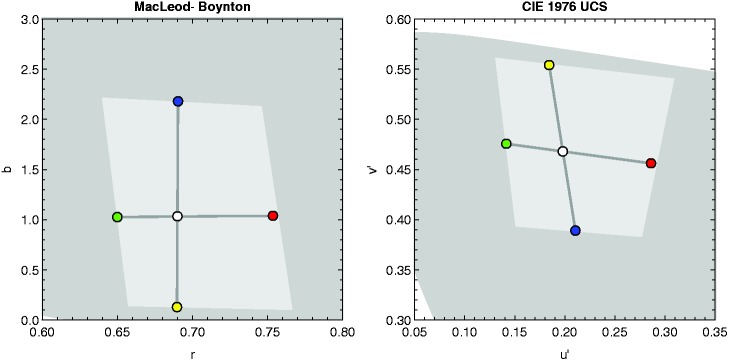
The chromaticity coordinates of the five base chromaticities used to construct the five different color conditions (see Figure 5) are shown both in the MacLeod-Boynton chromaticity diagram (MacLeod & Boynton, 1979) and the CIE 1976 (u', v') -UCS chromaticity diagram. The central achromatic point has the chromaticity of D65. The remaining chromatic points were grouped to two pairs of colors (red-green and blue-yellow). The chromaticities of these colors were chosen such that they were close to the gamut of the monitor at a luminance of 50 cd/m^2^ (represented by the light gray polygon).

The five color conditions were each related to a specific line segment in color space. The line segments were constructed from five base chromaticities: Beside an achromatic base color (at the chromaticity of the daylight equivalent D65), four additional chromaticities were chosen at the maximum distance to the achromatic point that was realizable inside the gamut of the monitor for a luminance of 50 cd/m^2^ (see Figure 4).

**Figure 5.**
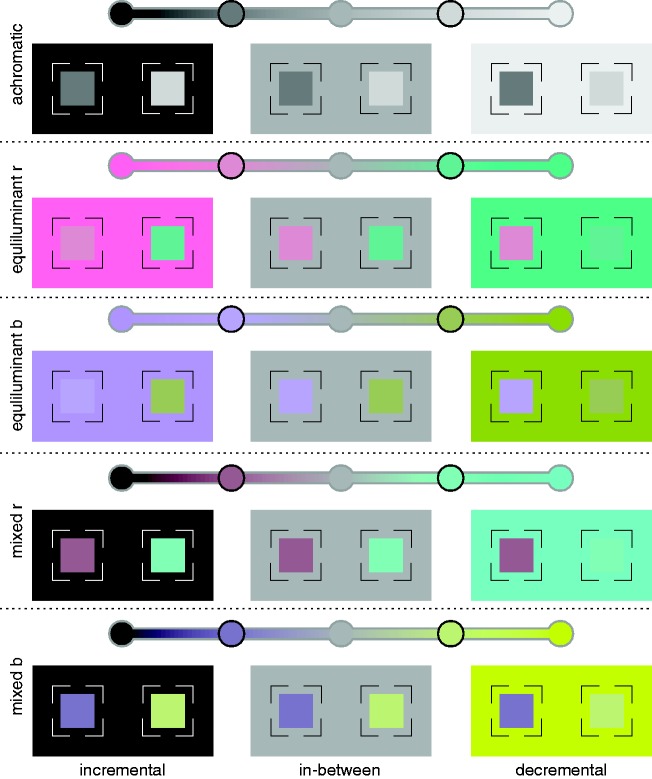
Schematic representation of the five different color conditions (rows) as they were examined in Experiment 1, each with its three different background conditions (columns). The top panel in each of the five sections schematically depicts the respective line segment in color space that is given by two endpoint colors (see Figure 4). The achromatic midpoint color is identical for all five color conditions (D65 with a luminance of 25 cd/m^2^). The three different background conditions for each color condition were determined by the midpoint color (“in-between” background condition) and the two endpoint colors (“incremental” and “decremental” background condition, respectively, see the bottom panel in each section). The colors of the center patches were also located on the same line segment in color space (disks with black border, here as an example with a binocular color contrast of 0.5). In the adjustment task of Experiment 1, the binocular color contrast between the monocular center patches could be interactively manipulated by the subjects (see Figure 6(a)).

The first color condition was given by a line segment in the u'v'L-color space (Wyszecki & Stiles, 1982) through the achromatic point and endpoints at 0 cd/m^2^ and 50 cd/m^2^ (top section in Figure 5). The four nonneutral chromaticities formed the endpoints of two line segments that were parallel to lines corresponding to the S and the L-M axis of the MacLeod-Boynton chromaticity diagram (MacLeod & Boynton, 1979; see left diagram in Figure 4). This way the S and L-M cone excitations could be varied in isolation in some stimulus conditions. Two equiluminant color conditions were defined by combining these line segments in the chromaticity diagram with a constant luminance of 25 cd/m^2^. The lines through the red and green chromaticities and through the blue and yellow chromaticities are referred to as the equiluminant *r* and equiluminant *b* condition, respectively (see Figure 5). In the mixed *r* condition, the green chromaticity was combined with a luminance of 50 cd/m^2^ and the red chromaticity with one of 0 cd/m^2^. In the mixed *b* condition, the yellow chromaticity was combined with a luminance of 50 cd/m^2^ and the blue chromaticity with 0 cd/m^2^ (Figure 5). The line segments corresponding to the five color conditions intersect at the central white point (D65) at 25 cd/m^2^ (see Figures 4 and 5).

Each of these color conditions was combined with three background conditions, referred to as incremental, decremental, and in-between. The colors of the incremental and decremental backgrounds were determined by one of the endpoint colors in each color condition (see Table 1, Figure 5). In the in-between condition, the same achromatic background color with a luminance of 25 cd/m^2^ was used in all five color conditions (middle column in Figure 5). The labeling of the background condition refers to the sign of the contrast between center and surround: If the LMS color code of the surround is subtracted from that of the center patch, then the resulting contrast vector can have any combination of positive and negative signs (cf. Mausfeld & Niederée, 1993). The last column of Table 1 shows the contrast codes of the two half-images resulting in the three background conditions. In the incremental and decremental conditions, these contrast codes had always equal signs. In the in-between background condition, they generally had opposite signs, that is, the contrast polarities were reversed between the two monocular half-images of the same stimulus. This means that the stimuli in the latter case fulfilled the inc-dec condition, which according to Anstis (2000) should produce strong lustrous impressions. Note that for the equilumiant *r* color condition, the labels “incremental” and “decremental” are somewhat misleading, because the contrast polarities were generally mixed across the three different color channels. In the mixed *b* condition, there is also the peculiarity that under the in-between background condition, there is no reversal of contrast polarity in the S-channel but only in the L- and M-channels (see Table 1).

**Table 1.** For Each of the Five Color Conditions, the Color Coordinates for the Different Background Conditions Are Shown.

Background condition	u'v'L	LMS	Contrast polarities						
Color condition: achromatic									
Incremental	(0.198, 0.468, 0)	(0, 0, 0)	+∘	+∘	+∘		+∘	+∘	+∘
In-between	(0.198, 0.468, 25)	(18.946, 8.514, 28.377)	-∘	-∘	-∘		+∘	+∘	+∘
Decremental	(0.198, 0.468, 50)	(37.892, 17.028, 56.753)	-∘	-∘	-∘		-∘	-∘	-∘
Color condition: equiluminant *r*									
Incremental	(0.287, 0.456, 25)	(21.007, 6.869, 28.965)	-∘	+∘	-∘		-∘	+∘	-∘
In-between	(0.198, 0.468, 25)	(18.946, 8.514, 28.377)	+∘	-∘	+∘		-∘	+∘	-∘
Decremental	(0.141, 0.476, 25)	(17.681, 9.522, 27.909)	+∘	-∘	+∘		+∘	-∘	+∘
Color condition: equiluminant *b*									
Incremental	(0.211, 0.388, 25)	(19.364, 8.683, 61.126)	-∘	-∘	-∘		-∘	-∘	-∘
In-between	(0.198, 0.468, 25)	(18.946, 8.514, 28.377)	+∘	+∘	+∘		-∘	-∘	-∘
Decremental	(0.184, 0.555, 25)	(18.631, 8.383, 3.473)	+∘	+∘	+∘		+∘	+∘	+∘
Color condition: mixed *r*									
Incremental	(0.287, 0.456, 0)	(0, 0, 0)	+∘	+∘	+∘		+∘	+∘	+∘
In-between	(0.198, 0.468, 25)	(18.946, 8.514, 28.377)	-∘	-∘	-∘		+∘	+∘	+∘
Decremental	(0.141, 0.476, 50)	(35.362, 19.043, 55.817)	-∘	-∘	-∘		-∘	-∘	-∘
Color condition: mixed *b*									
Incremental	(0.211, 0.388, 0)	(0, 0, 0)	+∘	+∘	+∘		+∘	+∘	+∘
In-between	(0.198, 0.468, 25)	(18.946, 8.514, 28.377)	-∘	-∘	+∘		+∘	+∘	+∘
Decremental	(0.184, 0.555, 50)	(37.262, 16.766, 6.947)	-∘	-∘	+∘		-∘	-∘	+∘

*Note.* Both in the CIE 1976 (u',v') UCS system (including luminance L) and in cone excitations LMS (Stockman, MacLeod, & Johnson, 1993, for 2°). The incremental and the decremental color codes also represent the endpoints of the line segment that was used to determine the colors of the center patches under each color condition (see Figure 5). The last column schematically shows the relationship of the contrast polarities for each color channel between the two monocular half-images of the stimuli under each background condition. A reversal of contrast polarities between the two eyes only occurs under the in-between background condition.

**Figure 6.**
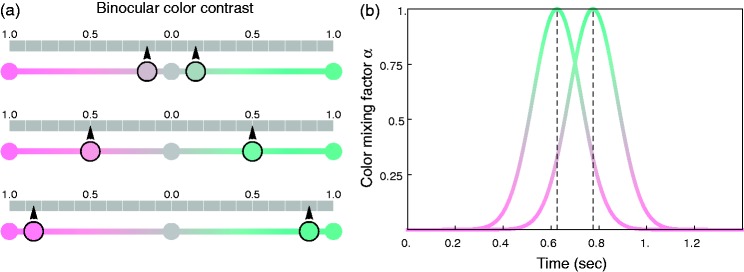
(a) Illustration of how the two monocular center patch colors for our static stimuli were derived from three different example values for the binocular color contrast *c* (0.15, 0.5, and 0.85 from top to bottom). The contrast value determines the relative position of the two patch colors between the central achromatic point and the two end points of the respective color line element (see Figures 4 and 5). (b) Construction of the dynamic stimulus: For the left and the right eye of the observer, the temporal weighting functions for the mixing factor α are shown (here, as an example, for the equiluminant *r* condition where the temporal variations in the colors of the two monocular center patches are schematically shown for a binocular color contrast of 0.85). The two colors from the static case determine the boundaries of the color variation, that is, the baseline and the peak of the Gaussians.

The color coordinates **p**^1^ and **p**^2^ of the left and right center patches of the stereoscopic stimuli were always located on the same line segment in color space defined by the respective color condition. The color contrast between **p**^1^ and **p**^2^ was varied by way of a parameter *c*, 0 ≤ *c* ≤ 1, which controlled the convex mixture between the achromatic point **w** = (**w**_u'_, **w**_v'_, **w**_L_)^T^ at 25 cd/m^2^ and the endpoints **e**^1^ and **e**^2^ of the corresponding line segment (see Figure 6(a)): **p**^i ^= *c***e**^i ^+ (1−*c*) **w,** for *i* = 1, 2. Note that the mixture is done separately in chromaticity space and luminance and not in a three-dimensional color space. This ensures that the mixed condition is a simple combination of the equiluminant and the achromatic condition.

In the classic static stimuli, the colors of the two center patches are constant during each trial (**p**^1^_static_ = **p**^1^ and **p**^2^_static_ = **p**^2^). In the dynamic counter-modulation stimuli, the colors of the two center patches were temporally modulated. At each time *t* during the stimulus presentation, the colors **p**^1^_dynamic_ and **p**^2^_dynamic_ were convex mixtures of the two original colors **p**^1^ and **p**^2^, where the mixing factors α_i_(*t*) followed a temporal Gaussian function (see Figure 6(b)): **p**^i^_dynamic_ = α_i_(t) **p**^1 ^+ (1−α_i_(t)) **p**^2^.

The Gaussian weighting function had a width of 100 ms and was scaled to a range between 0 and 1. The Gaussians were temporally shifted by 150 ms between the two half-images. In our first experiment, they were presented within a time window of 1400 ms (see Figure 6(b)). The peak of the functions was reached after 625 ms in one eye and after 775 ms in the other. In Experiments 2 and 3, the duration of the time window was reduced to 600 ms (with peaks at 225 and 375 ms, respectively). The narrower time interval was due to the fact that constant portions of the Gaussians were cut off on the left and right side. The reason for this modification was that Experiments 2 and 3 involved the comparison of the lustrous impressions between different stimuli, and we wanted to avoid that the subjects base their judgment on stimulus features other than the lustrous impression, for instance on the relative intensities of the baselines of the Gaussians.

During each trial of the experiments, the stimuli were presented as long as the subjects needed to make their decisions. For dynamic stimuli, this means that they were immediately repeated after the time window ended, resulting in a seamless sequence of cycles until the subjects fulfilled their task.

## Experiment 1—Contrast Threshold for Lustrous Impressions

The aim of Experiment 1 was to determine the absolute threshold for perceived luster in each stimulus condition, that is, the minimum binocular contrasts evoking an impression of luster. It was also taken into account that in some conditions, a lustrous impression cannot be achieved. In addition to the two presentation modes “static” and “counter modulation,” a further dynamic presentation method was used, which served as a control. This additional condition differed from “counter modulation” only in that there was no temporal peak separation between the two monocular intensity functions, that is, both eyes saw identical temporal color functions. In this way, we tested whether the presence of counter modulation is indeed a necessary condition for triggering a gloss impression in the dynamic case or whether such an impression can be produced by mere temporal color changes alone.

Each of the 45 stimulus conditions (5 Color Conditions × 3 Background Conditions × 3 Presentation Methods) was presented four times, resulting in a total of 180 trials that were presented in random order. The task of the subjects was to adjust the color contrast between the center patches of the two monocular half-images and to find the contrast at which a lustrous impression was just noticeable. The subjects were asked to deliberately oscillate around the contrast value that they initially set as the absolute threshold, in order to possibly find an even better setting. The adjustments were made with the “left” and “right” arrow keys of the keyboard. If the subjects failed to find a contrast value at which they perceived luster, they should mark the respective stimulus as “not lustrous” by use of the “up” or “down” arrow key. As a feedback, “not lustrous” was then displayed on the screen below the test stimulus, replacing the default message “lustrous.” In each trial, the test stimulus was presented together with an additional matte reference stimulus that always had a constant and identical color for both patches of the two half-images (D65 at 20 cd/m^2^ with binocular color contrast of 0.0). A comparison of the test stimulus with the matte anchor stimulus facilitated the detection of weak impressions of luster in the test stimulus. The test stimulus was always presented above the reference stimulus on the screen with a center-to-center distance of 9.15° of visual angle.

After the subjects had made their settings, they pressed the “return” key to move to the next trial. A dark adaptation interval of 3 seconds was inserted between trials.

If an experimental task requires difficult judgments, subjects sometimes use secondary stimulus features as a criterion instead of the perceptual criterion that is actually demanded. In the present experiment, for instance, the subjects could refer to the interocular color difference between the center patches rather than the lustrous impression and this could invalidate the threshold measurement. We aimed to prevent this by carefully instructing the subjects to use only the impression of luster as criterion. This instruction was first given in written form. In addition, the subjects had to complete a set of eight example stimuli prior to the experiment while the instructor was present. During this training session, the subjects were first asked to describe their impressions while freely manipulating the binocular color contrast between the two monocular center patches. We used this procedure to introduce the subjects to the phenomenon of binocular luster, since most of them were unfamiliar with it. When the subjects started to report something like a shimmering, shiny, glossy, lustrous, or similar appearance, they were told that this is the perceptual criterion to be used in the present task. This way we could also ensure that the subjects were able to haploscopically combine the two monocular half-images. Furthermore, the rating task at the end of each trial additionally served as a reminder to use only the lustrous appearance for a judgment of the stimuli.

### Subjects

Seven subjects took part in all three experiments who all had normal color vision as tested by means of the Ishihara plates (Ishihara, 1967), one of them being an author of the present article (G. W.). Five of the subjects were females, two were males, and their age was between 18 and 46 years (median = 23). All experiments of this study were carried out in accordance with the Code of Ethics of the World Medical Association (Declaration of Helsinki), and informed consent was obtained for experimentation with human subjects.

### Results

**Figure 7.**
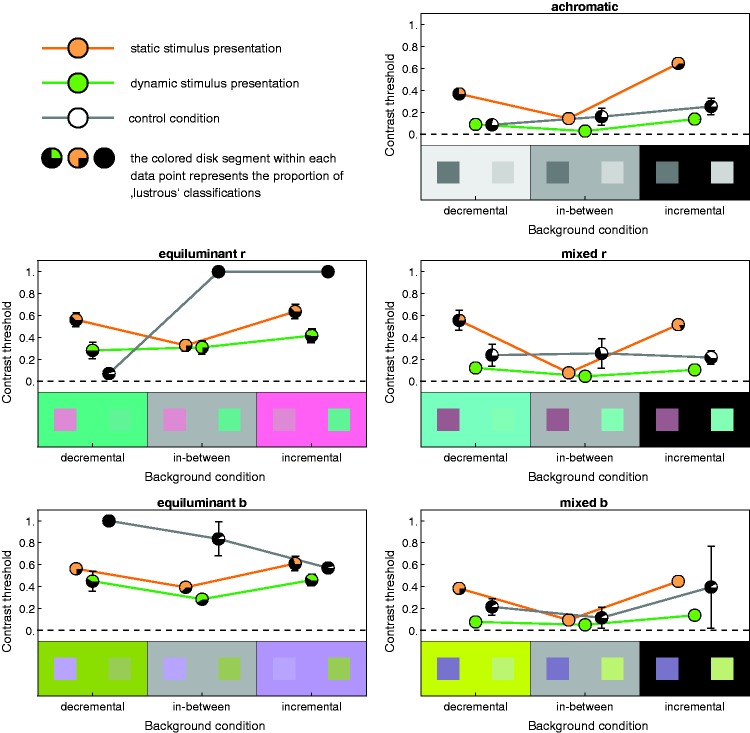
For each of the five color conditions (diagrams), the average settings of six subjects are shown, separated by the different presentation methods (green and orange lines; the achromatic curves show the settings of the control condition) and background conditions (horizontal position of the data points). The colored disk segment within each data point shows the relative proportion of stimuli that were perceived as lustrous. Error bars refer to ± *SEM*.

Figure 7 shows the results of the experiment, averaged across six of the seven subjects. The data of one subject were excluded since there were very few “lustrous” classifications, even in conditions where the stimuli were judged as “lustrous” in 100% of the cases by the remaining subjects, and the threshold settings for those stimuli that were perceived as lustrous were also extraordinarily high. Each of the graphs refers to one of the five color conditions. Within each graph, the results for the static, the dynamic, and the control condition are displayed in different colors. The horizontal positions of the data points refer to the three different background conditions. Each data point represents the mean contrast setting across the six subjects, after excluding settings that were marked as “not lustrous.” The relative proportion of the settings that were perceived as lustrous is displayed as a colored disk segment within each data point.

The most salient feature is the obvious similarity in the trends of the absolute threshold settings in all color conditions that included luminance variations (see the plots in the right column in Figure 7). This means that the additional variation in chromaticity in two of these conditions has almost no effect.

#### Dynamic stimuli with luminance variation

In these conditions, the thresholds obtained with dynamically presented stimuli were in general rather small (with mean values between 0.028 and 0.138) and much lower than those found in the static case. The absolute differences between the background conditions were small, but some of them were nevertheless statistically significant (a one-way analysis of variance [ANOVA] performed on each of the three sets of dynamic stimuli with luminance variations revealed significant differences in all three cases: for the achromatic condition, *F*(2, 68) = 36.63, *p* < .001, for the mixed *r* condition, *F*(2, 69) = 10.16, *p* < .001, and for the mixed *b* condition, *F*(2, 69) = 16.26, *p* < .001). A Bonferroni post hoc test revealed that in both the achromatic and the mixed *b* color condition, the mean settings under the incremental condition differ significantly from those under the decremental and the in-between conditions, whereas in the mixed *r* color condition, the means in the in-between condition differ significantly from those of the remaining two background conditions. Furthermore, the dynamic stimuli appeared lustrous almost throughout.

#### Static stimuli with luminance variation

The luster perceived in statically presented stimuli (orange data curves in Figure 7) depended much stronger on the background conditions: While the contrast values in the in-between condition were also low (with mean values between 0.077 and 0.143), considerably higher values were obtained in the remaining two background conditions (with mean values ranging from 0.368 to 0.647). A large number of the stimuli in the incremental and decremental conditions were judged as not lustrous at all. The proportion of lustrous impressions was particularly low in the decremental conditions (40.3% on average compared with 81.94% in the incremental conditions).

#### Equiluminant stimuli

In the two equiluminant color conditions, it is much more difficult to identify a clear trend in the data, as there were very strong differences between the subjects, which is why the respective data plots showing the mean contrast settings across subjects (left column in Figure 7) do not provide a representative picture: The percentage of “lustrous” classifications ranged from 8.33% (i.e., 4 of 48 stimuli) to 95.8% with an average of 54.43% for the six subjects. These subjects rated 52.78% of the static equiluminant stimuli and 52.08% of the counter-modulation stimuli as “lustrous.” Usually, stimuli in the in-between background condition received more “lustrous” classifications than those in the decremental and incremental conditions. With the exception of one subject, the subjects chose slightly lower contrast thresholds in the dynamic case. The background conditions generally had a much lower and less systematic impact on the threshold settings than in the color conditions with luminance variation.

#### Control stimuli

Significant differences between subjects also occurred in the control condition: With only 1 or even 0 “lustrous” classifications for the total set of 60 control stimuli (i.e., 1.67% and 0%, respectively), the majority of the subjects (four out of six) judged these stimuli as nonlustrous. However, two of the subjects judged these stimuli as “lustrous” in 33.3% and 35% of the cases, respectively. The chance that a control stimulus was judged as lustrous was in stimuli containing luminance variations about six times as high than in equiluminant stimuli. There was also a slight preference to see luster in control stimuli that additionally varied along a red-green chromaticity axis.

## Discussion

The results obtained with the classical static presentation method generally confirm the findings reported in Anstis (2000): A vivid and stable impression of luster could only be evoked if a spatial increment was binocularly combined with a spatial decrement (i.e., when the two respective half-images had reversed contrast polarities, as it was realized in the in-between background condition). This is particularly pronounced in stimulus conditions that included luminance variations (right column in Figure 7). Our finding that an additional variation along a chromatic axis does not have any systematic effect on the thresholds (see the mixed *r* and the mixed *b* condition in Figure 7) suggests that the chromatic content is either ignored or, to some part, even “overlooked” by the visual system: As Jennings and Kingdom (2016) have found, the sensitivity to detect chromatic differences between eyes is reduced when in addition luminance contrasts are present. In the equiluminant conditions, the respective thresholds were considerably higher, but lustrous sensations were nevertheless obtained in the majority of the cases.

Compared with the static stimuli, the counter-modulation stimuli were less dependent on the background conditions, which influence the contrast polarities. As long as the stimuli contained luminance variations, the counter-modulation stimuli were reliably perceived as lustrous. In the equiluminant color conditions, in which the stimuli were less physically plausible, the rate of lustrous impressions was considerably lower, at least on average.

These results seem to be largely in line with a conflict explanation of the static case and the functional interpretation of the counter-modulation stimulus.

Furthermore, the results obtained with the control condition, that is, with dynamic stimuli that lack counter modulation generally indicates that counter modulation is a crucial feature to produce a reliable impression of luster, especially for those stimuli that include variations in luminance. However, the fact that two of our subjects judged also some of the control stimuli as lustrous suggests that temporal variations in luminance alone may be sufficient to evoke perceived luster. One may speculate that the control stimuli had a similar effect as the monocular flicker stimuli that Anstis (2000) found to be suitable to produce the perception of luster. However, while Anstis used a flicker frequency of 16 Hz, our control stimuli had a temporal distance of approximately 350 ms between the baseline and the peak of the temporal color function (or the darkest and the brightest point of the function, respectively, see Figure 6(b)), which is equivalent to a frequency of about 2.86 Hz. In addition, Anstis (2000) found that the lustrous effect of his flicker stimuli, as well as his static stimuli, strongly depends on the luminance of the surround, that is, on the presence or absence of reversed contrast polarities between eyes. Translated to our experimental design, this would mean that the data curves representing the control condition in Figure 7 should be similar in shape to the curves representing the static presentation method, which is not the case, because the results obtained with the control stimuli show no systematic dependence on the background condition.

## Experiment 2—Equidistant Scale for Perceived Luster

In the second experiment, we investigated the quantitative relationship between binocular color contrast and the strength of perceived luster. The purpose of this scaling experiment was twofold: On the one hand, we wanted to examine whether the shape of the scaling curve depends on the presentation method (i.e., static vs. dynamic). On the other hand, we aimed to construct a perceptually equidistant luster scale that could be used in the matching task that was employed in Experiment 3.

There are a number of related studies that aim to establish a perceptual glossiness scale (Billmeyer & O’Donnell, 1987; Ferwerda, Pellacini, & Greenberg, 2001; Harrison & Poulter, 1951; Obein, Knoblauch, & Viénot, 2004). These studies differ with respect to the kind of gloss samples, objective gloss measures, and experimental methods they used. Obein et al. (2004), for instance, defined a physical gloss measure for real surfaces and then determined a psychophysical scale for perceived gloss. Our approach is different in that the physical dimension is not directly related to surface properties but to binocular contrasts and that the perceptual dimension is binocular luster, that is, a phenomenon where it is not yet clear whether and, if so, how it is related to the perception of surface gloss.

In the scaling experiment, we used MLDS (“maximum likelihood difference scaling”) as proposed by Maloney and Yang (2003; see also Knoblauch & Maloney, 2008). This method is based on comparisons of the perceived differences in two pairs of stimuli that objectively differ in a certain attribute. In our case, we used pairs (A, B) and (C, D) of stereoscopic luster stimuli, where each stimulus pair differed in binocular color contrast. The same two presentation modes (static vs. dynamic) as in Experiment 1 were used, but only the combination of the “in-between” background condition with the achromatic color condition was realized. These were the conditions where the smallest absolute thresholds were observed which implies a large range of binocular contrasts leading to perceptual luster. We tested 11 different contrast values. The minimum value was 0.04 (which was sufficient to make the center patch discernable from its surround), and the remaining values ranged from 0.1 to 1.0 in steps of 0.1. The combination of contrast values for each quadruple of stimuli (A, B, C, and D) was restricted by the method of nonoverlapping quadruples (cf. Knoblauch & Maloney, 2008), where the respective contrast values *c*_i_ meet the requirement *c*_A_ < *c*_B_ < *c*_C_ < *c*_D_. With 11 different contrast values, this leads to 330 different nonoverlapping stimulus quadruples that were presented in random order during the experiment.

**Figure 8.**
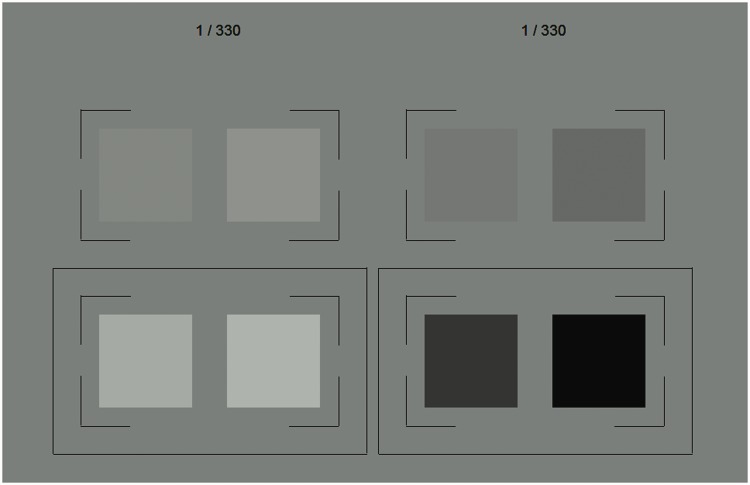
Screenshot of the center area of our display during the second experiment showing a static stimulus quadruple. The two pairs of stereoscopic stimuli that had to be compared by the subjects are displayed one above the other. The two stimuli of each pair are presented side-by-side in the fused percept (for instance, the leftmost patch in the top row represents the left half-image of Stimulus A while the third patch in this row represents its corresponding right half image; accordingly, the second and the forth patches represent the left and right half-images of Stimulus B, respectively). The subjects made their decision by using the arrow keys of the keyboard, whereafter the selected pair was marked with a rectangular frame.

During each trial, the two pairs of stimuli were displayed simultaneously on the screen, one above the other with a vertical center-to-center distance of 7.44° of visual angle. For each pair of these stereoscopic stimuli, the two center patches of each monocular half-image were presented side by side with a horizontal distance of 5.15° of visual angle (see Figure 8).

The static and dynamic presentation methods were tested in different sessions. In each trial, the subjects had to select that stimuli pair in which the difference in the strength of perceived luster appeared larger. The subjects used the “up” and “down” arrow keys to select and as feedback a one-pixel thick frame was drawn around the selected pair (see Figure 8). The subjects pressed the return key to confirm their decision. The next trial started after an adaptation period of 3 seconds during which the colors of all center patches of the display were set to the background color (D65 at 25 cd/m^2^). Again, there was no time restriction for the stimulus presentation, so that the subjects could use as much time as they needed to perform the task. As part of the instruction, the subjects had to complete a set of six different example stimuli prior to each of the two sessions while the instructor was present.

### Results

**Figure 9.**
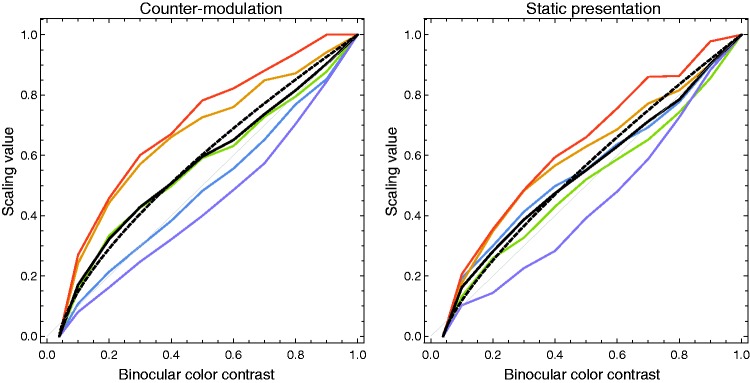
For the dynamic (left diagram) and the static presentation method (right diagram), the scaling curves for the individual subjects (colored lines) as well as the averaged values (black line) are shown. The dashed black line represents the fit of the mean curve with a power function.

Figure 9 shows the results of the scaling experiment for the static and the dynamic presentation methods. In both diagrams, the scaling curves for five of seven subjects are shown as colored lines. The average scaling values are shown as black lines together with a dashed black line that represents the fit of these averaged values with a power. The data from two of the seven subjects were excluded, since they produced quite extreme, partially nonmonotonic scaling curves whose shapes were either hard to fit with a power function or whose exponents were far from the general trend (for instance, one subject’s data for the static condition had an exponent of 0.187 while the exponent of the other subject was 3.136). The scaling values were calculated with the MLDS package for R provided by Knoblauch and Maloney (2008). Note that in Figure 9, only the course of the curves between the static and the dynamic condition can be compared. The MLDS procedure does not provide information about absolute values, and all curves are therefore normalized to a values range from 0 to 1. A direct comparison of the relative strengths of perceived luster between the different conditions is the subject of Experiment 3.

A comparison of the two diagrams in Figure 9 indicates that the two presentation methods lead to scaling curves that differ only slightly with respect to the exponent of the fitted power functions (a Wilcoxon signed-rank test performed on the two related sets of individual exponents revealed a nonsignificant difference with *p* = .59). For static presentation, the fitted average curve is slightly closer to a linear function (with an exponent of 0.77) than the one with dynamic presentation (with an exponent of 0.689). The ranges of the exponents of the individual fit functions are also comparable between the presentation methods (between 0.549 and 1.258 for static presentation and between 0.41 and 1.232 for the counter-modulation stimuli).

However, despite these similarities in the scaling data, the reports of the subjects suggest some fundamental differences with respect to the stability of perceived luster between the static and the dynamic presentation method. Most of the subjects (five of the seven subjects that originally took part in the experiment) noted that with static stimuli, they experienced strong binocular rivalry, that is, the differently colored center patches of the two monocular half-images seem to compete for perceptual dominance in the fused percept (see Blake & Logothetis, 2002). The dynamic stimuli, in contrast, were generally experienced as stable and easy to fuse, despite the fact that the lustrous impression only occurred during the brief counter-modulation phase of 150 ms (see Figures 2 and 6(b)). Only two of the subjects felt somewhat irritated by the flashing character of these stimuli.

### Discussion

The scaling data obtained with the two different presentation methods show that the perceived luster induced by both types of stimuli depends in a similar way on the contrast between the centers of both half-images. This observation suggests a similarity in the responsible processes. It is at present unclear, how the interfering effect of binocular rivalry, which is only found with statically presented stimuli, should be interpreted. It could be an independent process specific for static stimuli that is not directly related to the perception of luster. Alternatively, it may point to more fundamental differences between the processes responsible for the impression of luster in static and dynamic stimuli.

Another reason for the lack of binocular rivalry in our counter-modulation stimuli could be that there are different integration times for luster and rivalry: In the dynamic stimuli, the lustrous impression only occurs during a small time period within each 600 ms lasting cycle which is located between the peaks of the two monocular temporal color functions (Mausfeld et al., 2014, see the shaded area in Figure 2). In the present study, this time interval was 150 ms which seems sufficient to evoke the impression of luster (Formankiewicz & Mollon, 2009; Ludwig, Pieper, & Lachnit, 2007). However, for binocular rivalry to come to awareness, which is characterized by an alternating perception of the two different monocular inputs, this interval of 150 ms might be too short (Blake & Logothetis, 2002).

Following the suggestions of one of the reviewers, we tried out a different kind of temporal color function without a temporal offset between counter-modulation phases (i.e., the stimulus comprised a seamless sequence of counter-modulation phases). Instead of Gaussians, we used sinusoidal functions with a wavelength of 300 ms which were presented in antiphase to the two eyes. As an informal result, we actually found that compared with our original counter-modulation stimuli, the new version appeared a slightly more unsteady while the lustrous impression seemed to be unaffected. Compared with the static stimuli, though, the degree of binocular rivalry was rather marginal. However, there is another aspect in our counter-modulation stimuli that differs from the static ones and that may also have contributed to the greater stability of the dynamic stimuli: Even within the 150-ms time interval of counter modulation, the binocular color contrast is not constant but varies continuously from maximum to zero (at the crossing point between the two monocular color functions) to maximum with swapped colors between eyes (see Figure 6(b)).

## Experiment 3—Determining the Strength of Perceived Luster

In Experiment 3, we used a matching task to compare the relative strength of perceived luster in static and dynamic stimuli under comparable conditions. The comparison was done indirectly, by matching both the static and dynamic test stimuli for each of several context conditions by an adjustable stimulus (“comparison stimulus”) presented in a fixed reference condition.

The context conditions included two of the five color conditions already used in Experiment 1, namely, the mixed *r* condition (as a representative of the three conditions that include luminance variations) and the equiluminant *r* condition (as a representative of the two equiluminant conditions). For both color conditions, all three background conditions were tested (i.e., “incremental,” “decremental,” and “in-between”). The binocular color contrasts in the test stimulus were varied in three steps by setting *c* to 0.6, 0.75, or 0.9, with the lowest value of 0.6 being approximately equal to the highest contrast value for the absolute threshold for luster found in Experiment 1 under the chosen conditions (see Figure 7). This resulted in 36 different stimuli (2 Presentation Modes × 2 Color Conditions × 3 Background Conditions × 3 Binocular Color Contrasts). The setting for each stimulus was repeated four times. The total set of 144 trials was presented in random order during the experiment.

The dynamic comparison stimulus was presented in the achromatic color condition and the in-between background condition, because in Experiment 1, this was the combination of conditions for which the lowest absolute threshold for luster was found. We therefore expected this combination to allow the largest possible variation in the strength of perceived luster.

During each trial, the test and the comparison stimulus were displayed simultaneously, one above the other. Because the backgrounds of the test and comparison stimuli may be of different color, the entire screen was split in halves along the vertical axis. We balanced the vertical position of the test and the comparison stimulus such that in half of the trials, the test stimulus was presented in the top half while the comparison stimulus was shown in the bottom half and vice versa. The center patches of the two stimuli were presented with a vertical center-to-center distance of 12.89° of visual angle.

The subjects were asked to match the perceived luster in the test and the comparison stimulus as closely as possible by adjusting the binocular luminance contrast in the comparison stimulus with the left and right arrow keys. The results of Experiment 2 were used to establish an approximately equidistant luster scale for the comparison stimulus. To this end, the original contrast values were transformed by a power function with exponent 1/0.76 (see Experiment 2). If a given test stimulus did not evoke a lustrous impression at all, the subjects should indicate this by pressing a corresponding key. Once the subjects completed a trial, they confirmed their settings with the return key. The next trial started after a dark adaptation period of 3 seconds. The start value for the binocular luminance contrast of the comparison stimulus was chosen randomly from the interval [0, 1] in each trial. As in Experiments 1 and 2, the subjects had to complete a set of five example stimuli under the supervision of the instructor before the experiment was started.

### Results

**Figure 10.**
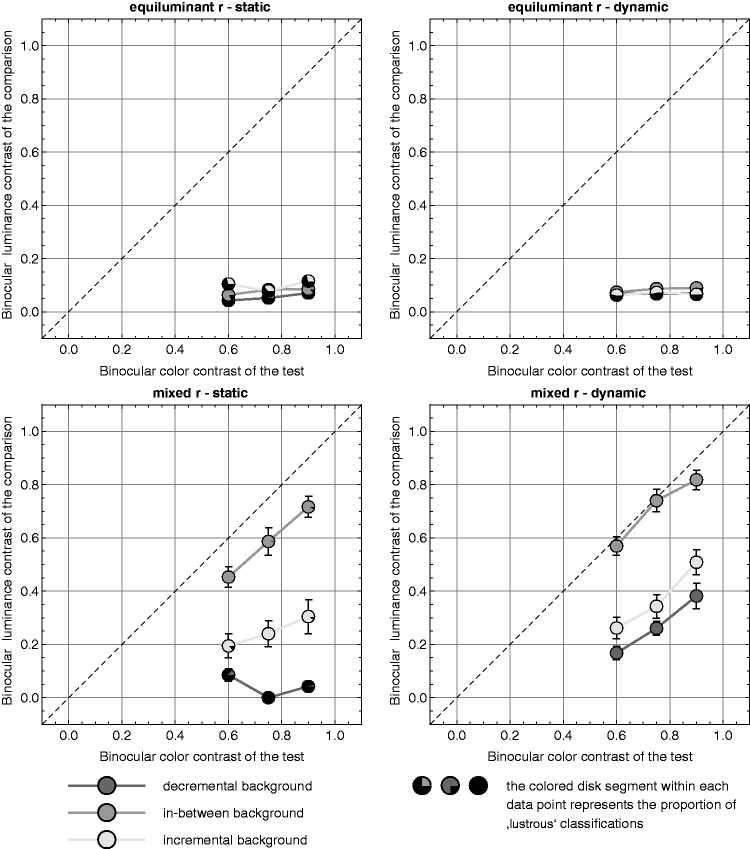
Mean contrast settings of the comparison stimulus across all seven subjects plotted against the contrast values of the test stimuli for all three background conditions. The rows contain the plots for the two different color conditions, the columns for the two presentation methods. The error bars represent ± *SEM*.

Figure 10 shows the results obtained in the two color conditions (rows) and the two presentation methods (columns). In each diagram, the mean settings of the contrast values in the comparison stimulus are plotted against the contrast values in the test stimuli for all three different background conditions (lines in each diagram). Each data point represents the average settings of the seven subjects in test stimuli that they had judged as lustrous. The relative proportions of test stimuli that were classified as lustrous are shown by the size of the colored disk segments within a data point.

In general, the results confirm the trends we found in Experiment 1 (see Figure 7). A look at the relative proportions of test stimuli classified as lustrous suggests that lustrous appearances were only reliably evoked under the conditions in which luminance variations were present (mixed *r*, see bottom row in Figure 10): In all background conditions with dynamic stimuli and in the “in-between” condition with static stimuli, more than 95% of the cases were judged to evoke perceptual luster (note that six of the seven subjects actually had a rate of 100% for these four conditions, the remaining subject again reported strong rivalry effects with the static stimuli). This indicates that the contrast polarities between center and surround had a strong influence on perceived luster in static but not in dynamic stimuli (compare the corresponding diagram in Figure 7). While on average, the subjects judged 96.4% of the static stimuli to be lustrous when they had reversed contrast polarities (i.e., in “in-between” background condition), this proportion dropped slightly to an average value of 91.7% in the incremental condition and dropped to an average value of only 5.95% in the decremental condition.

With respect to the strength of the perceived luster in the mixed *r* conditions, a clear pattern can be seen: For each test contrast, the strength was maximal for stimuli with reversed contrast polarities (“in-between” background condition, see the mid gray curves in the bottom diagrams of Figure 10), clearly lower for the incremental background condition, and the weakest impressions of luster were always found in the decremental background condition. Due to the large number of low cell frequencies in the static case under the decremental background condition (bottom left diagram in Figure 10), we calculated a two-way ANOVA separately for the static and the dynamic case with the factors “background condition” and “test contrast,” where in the static case, the background level “decremental” was omitted. For both presentation methods, we found significant main effects for the factor “background” (in the dynamic case, *F*(2, 243) = 103.86, *p* < .001; in the static case, *F*(1, 156) = 71.99, *p* < .001) and the factor “test contrast” (in the dynamic case, *F*(2, 243) = 27.24, *p* < .001; in the static case, *F*(2, 156) = 7.59, *p* < .001). A Bonferroni post hoc test revealed significant differences between all levels of the factor “background” both between the three levels of the dynamic and between the two levels of the static presentation method.

Although the curves show the same order for the two presentation methods, they clearly differ in their absolute values: Under static presentation, the average contrast settings were considerably lower than for their dynamic counterparts (compare the corresponding curves between the two bottom diagrams in Figure 10). This is particularly true for the settings under the decremental background condition (dark gray curves in the respective diagrams in Figure 10), where in the static case, the stimuli even did not appear lustrous at all in 94% of the cases (79 out of 84 stimuli). We performed a three-way ANOVA on the combined sets of static and dynamic data of the mixed *r* color condition with the factors “presentation method,” “background,” and “test contrast,” again with the background level “decremental” excluded. We found significant main effects for all factors: for the presentation method with *F*(2, 314) = 22.09, *p* < .001, for the background with *F*(2, 314) = 170.48, *p* < .001, and for the test contrast with *F*(2, 314) = 24.12, *p* < .001.

In the equiluminant color conditions (top row in Figure 10), rather high proportions of lustrous appearances were observed in the in-between background condition with both static (83%) and dynamic (92.9%) presentation. For the two remaining background conditions with consistent contrast polarities, these proportions were considerably lower. For the static presentation method, the proportions of luster classifications were 25.0% and 35.7% (top left diagram in Figure 10) and for the dynamic stimuli 46.4% and 53.6% (top right diagram in Figure 10). However, the strength of perceived luster was generally very low for all equiluminant stimuli, and there was no clear dependence of the contrast settings on the background condition or even on the contrast value of the test stimulus.

### Discussion

The strong effect of the background conditions on the strength of perceived luster in dynamic stimuli (bottom right diagram in Figure 10) suggests that spatial contrast information also play a role in counter-modulation stimuli. If one assumes—as it is done in the Oppel-Helmholtz approach—that the visual system interprets the background as a diffusely reflecting part of the surface and the center as highlight, then the only physically plausible center-surround configuration is one in which both center patches are brighter than the surround. This could explain why the lustrous impressions under the “incremental” condition were stronger than in the “decremental” background condition. The fact, however, that the lustrous impressions were strongest under the “in-between” background condition is not compatible with this reasoning.

A possible solution to this inconsistency could be that an independent effect based on contrast polarity makes an additional contribution to perceived luster. This may also explain some of the findings in the equiluminant color condition, that is, a condition that was considered the least physically plausible one with respect to gloss, where also comparatively high proportions of luster classifications were found in the in-between conditions.

The results in the static presentation condition (left column in Figure 10) indicate that the perceived luster observed with these stimuli cannot be exclusively attributed to a mechanism that simply responds to any kind of a neuronal conflict, because one would then expect that the equiluminant and the mixed condition would lead to effects of similar strength in the in-between condition. This is clearly not the case. To explain the observed difference, one needs to additionally assume that the responsible mechanism is more responsive to conflicting luminance information rather than to other channel-wise conflicts.

Rather high proportions of the static stimuli with luminance variations were judged as lustrous in the incremental condition which does not produce any neuronal conflict in terms of reversed contrast polarities. This is not in line with the assumption that perceived luster depends solely on such conflicts. This suggests that even in the static case, contrast polarity is not the sole cause of perceived luster. It seems possible that physical plausibility plays also a role.

Also the fact that the lustrous impression was stronger under dynamic presentation compared with the static stimuli (at least when luminance variations were present, see the bottom diagrams in Figure 10) might be somewhat surprising: Due to the construction of the counter-modulation stimuli, the binocular color contrast was considerably lower at any point in time during the presentation compared with that of their static counterparts. While in the static case, this contrast had a constant magnitude, it varied over time in the counter-modulation stimuli, ranging between 0% and about 70% of the magnitude of the corresponding static stimuli (see the difference between the two curves in Figures 2 and 6(b)). From the perspective of a low-level mechanism that is based on interocular color differences (see, for instance, Formankiewicz & Mollon, 2009; Malkoc & Kingdom, 2012), one would generally assume a monotonic relationship between this interocular contrast and the magnitude of the lustrous response—which is what we have found in Experiment 2 (see Figure 9). Therefore, one would expect stronger responses in the static case. If, on the other hand, one considers the results of studies on the perception of surface gloss, it is a well-established finding that dynamic stimuli, for example, rotating objects under a fixed illumination are perceived as considerably glossier than static objects (Doerschner, et al., 2011; Hartung & Kersten, 2002; Sakano & Ando, 2010; Wendt & Faul, 2018).

## General Discussion

In this study, we investigated stereoscopic luster, that is, the phenomenon that certain pairs of simple two-dimensional stimuli that are used as half-images in a stereoscopic presentation can evoke a vivid impression of luster. The classic stimulus used to demonstrate this effect comprises a pair of fixed achromatic center-surround stimuli that only differ in the luminance of the center. For this type of stimulus, Anstis (2000) provided evidence in favor of a low-level explanation of the luster that is perceived in the center region: The observation that the effect did only occur when the luminances of the centers have a different contrast polarity with respect to the common surround luminance, led him to conclude that the lustrous impression is a side effect of the inability of the visual system to combine incremental and decremental contrast information.

Our investigation was mainly motivated by recent findings obtained with a dynamic variant of the classic stimulus, where the luminances of the two central patches are not static, but vary systematically with time (Mausfeld et al., 2014). The fact that with this stimulus luster was also seen without different contrast polarities challenged the low-level explanation. The reported finding instead provided evidence for the hypothesis that the perceived luster is the result of an interpretation of this stimulus as a dynamic, motion-induced cue for glossiness.

To investigate whether the mechanism underlying the effects observed with these types of stimuli are similar or different, we directly compared the perceived luster elicited by the classical static stimulus and the dynamic stimulus under comparable conditions. We varied the contrast relations of the central patches to the surround and the plausibility of an interpretation of the central patch as a highlight of a glossy surface, that is, stimulus properties, which play an important role in the low-level and the more high-level explanation, respectively.

The results obtained in three experiments are not as clear-cut as we expected. We indeed found evidence that supports the assumption that the perceived luster caused by static and dynamic stimuli are different phenomena that rely on different mechanisms, which seem to some extent compatible with the explanations proposed by Anstis (2000) and Mausfeld et al. (2014), respectively. However, the perceived luster in both types of stimuli was also partially affected by the kind of information that according to the proposed explanations should predominantly determine the effect in the other type. As a consequence, the result pattern obtained for both types of stimuli in all the experiments also show many similarities.

A common finding for both types of stimuli was that strong perceived luster does only occur in stimuli containing variations in luminance. This is in line with expectations derived from an interpretation in terms of a physical gloss situation. For the classical stimulus configuration, this finding suggests that a simple reversal of contrast polarities between the two monocular half-images is in itself not sufficient to generate strong percepts of luster. Apparently, the underlying mechanism is restricted to conflicts in the luminance channel and does not generalize to conflicts in cone-excitations. It might also be that this asymmetry is due to the fact that the absolute contrasts in our equiluminant stimuli were considerably weaker than those realized in stimuli that varied in luminance (see Table 1). However, Jung et al. (2013) found that purely chromatic stimuli were generally less able to produce gloss impressions than stimuli containing luminance variations, even if chromatic and luminance contrasts were made equally strong perceptually.

In support of the neuronal conflict explanation for the classical phenomenon of stereoscopic luster, we found that static stimuli without reversed contrast polarities were characterized by (a) comparatively high absolute contrast thresholds for perceived luster (Experiment 1), (b) a strongly reduced strength of perceived luster (Experiment 3), and (c) low proportions of classifications as lustrous (Experiments 1 and 3). However, there was one interesting exception from this rule: Stimuli with luminance variations, in which both monocular half-images were incremental relative to the background, were perceived as fairly lustrous. Since these two stimulus features are in line with physical regularities in ecological gloss situations, this could indicate that the underlying mechanism of the visual system combines different kinds of information rather than exclusively respond to conflicting contrast information.

The findings with dynamic stimuli in general differed considerably from the results obtained with static stimuli: Dynamic stimuli with luminance variations reliably evoked perceived luster at very low contrast thresholds in almost 100% of the trials, independent from spatial contrast information. Furthermore, the perceived luster appeared considerably steadier than that obtained with the static stimuli. This is indicated by the reports of the subjects, who often experienced binocular rivalry with static stimuli but not with dynamic stimuli. The matching experiment (Experiment 3) revealed that the *strength* of the perceived luster actually depends on spatial contrast information: In agreement with expectations derived from physical regularities found in glossy materials, the spatially incremental stimuli produced slightly stronger lustrous impressions than spatially decremental stimuli. But, contrary to what one would expect from this functional perspective, the strength of perceived luster was even higher when reversed contrast polarities were involved. This finding suggests that the mechanism responsible for luster in dynamic stimuli takes the compatibility with the physical regularities of ecological gloss situations into account but is not immune from low-level effects from neuronal conflicts.

In summary, we actually found systematic differences between the static and dynamic version of stereoscopic luster. However, the assumption that the luster evoked by static stimuli can be explained by a low-level process and the luster evoked in dynamic stimuli by a more high-level process referring to physical regularities in glossy materials seems too simple. Although these two explanations actually account for more aspects of the data in the situation for which they were proposed, there are also observations that do not fit. A possible solution for this finding could be that both processes are involved in each stimulus situation, but that the weight with which they contribute to perceived luster differs.

It would be interesting to see whether investigations from the field of brain research could throw some more light on this matter. Assuming that the classical phenomenon of stereoscopic luster results from a conflict at an early physiological level while counter modulation might be a regular cue in the perception of surface gloss, one may expect different brain regions along the visual pathway to be involved in the processing of the two stimuli. Using imaging techniques, researchers have already identified a number of cortical areas that seem to be related to the perception of glossy objects which include regions in V2, V3, V4, VO-1, VO-2, CoS, LO-1, and V3A/B (Wada, Sakano, & Ando, 2014) as well as in V3B/KO and in the posterior fusiform sulcus (Sun, Ban, Di Luca, & Welchman, 2015).

It is also possible that differences in the visual processing already take place at an early physiological level, where the two monocular signals are combined into different types of binocular channels (Henriksen & Read, 2016; Kingdom, 2012). There is a growing body of studies dealing with the detection of interocular differences in color or luminance (Formankiewicz & Mollon, 2009; Jennings & Kingdom, 2016; Malkoc & Kingdom, 2012; Meese, Georgeson, & Baker, 2006), some of them indicating that the underlying mechanisms are located at this level (Georgeson et al., 2016; Kingdom, Jennings, & Georgeson, 2018). In this context, phenomena as binocular luster and binocular rivalry, which could be different responses of the same mechanism, serve as cues signaling the presence of interocular differences (Georgeson et al., 2016; Jennings & Kingdom, 2016; Malkoc & Kingdom, 2012). From this view, it would not be necessary to assume different mechanisms for the joint occurrence of luster and rivalry, as it was found in the present study exclusively with static stimuli. One may rather ask whether the absence of binocular rivalry in our counter-modulation stimuli can be taken as an indication that such dynamic signals are processed in a different mechanism. However, as we have already pointed out in the discussion section of Experiment 2, our static and dynamic stimuli are hard to compare in this regard, since in the counter-modulation stimuli, the interocular color differences varied considerably over time, which may have contributed to a better fusion. The finding, however, that the counter-modulation stimuli were generally perceived as more lustrous compared with their static counterparts (see Figure 10), although—locally—they were characterized by considerably lower interocular color differences, seems to challenge the idea of a common mechanism that is based on such interocular differences in color or luminance.

It is also currently unclear whether the processing of different subtypes of stimuli takes place in a common or separate mechanism, namely, the processing of stimuli that are characterized by consistent or reversed contrast polarities between eyes. Whenever we referred to a conflict in the present context, we meant a conflict in terms of contrast polarities and our results do indeed suggest that the binocular combination of increments and decrements produces extraordinarily strong lustrous appearances (see Anstis, 2000). However, we also found noticeable lustrous responses with stimuli that comprised equal contrast polarities, especially with purely incremental stimuli, where a conflict only occurs with regard to the size but not to the sign of the contrasts (see also Formankiewicz & Mollon, 2009; Sheedy & Stocker, 1984). Recently, Georgeson et al. (2016) have proposed a model that takes different forms of binocular contrast discrimination into account, where binocular luster—as a cue for an interocular contrast difference—appears as one of the model components. In its current incarnation, this model predicts lustrous responses exclusively for stimuli with opposite contrast polarities. Our present findings, however, suggest that for a more complete model, that is, a model that represents a common mechanism, both forms of conflict would have to be integrated: While moderate lustrous impressions can already be elicited by an interocular color difference alone, this response will be boosted when in addition reversed contrast polarities are involved.
